# Prevalence and Pathogen Profiles of Yak Diarrhea in Ganzi Tibetan Autonomous Prefecture, Sichuan Province, China

**DOI:** 10.3390/pathogens15050552

**Published:** 2026-05-20

**Authors:** Youying Li, Xue Zhang, Yao Pan, Jianping Wu, Wenbin Xu, Zhaobin Xia, Songming Li, Deyun Wang, Lan Lan, Honghui Ren

**Affiliations:** 1 Key Laboratory of Ecological Protection and Characteristic Industry Cultivation in Hengduan Mountain Area at Sichuan Minzu College of Sichuan Provincial Department of Education and Ganzi Prefecture, Kangding 626001, China; 2 School of Ecology and Agriculture, Sichuan Minzu College, Kangding 626001, China; 3 College of Animal Science and Technology, Yunnan Agricultural University, Kunming 650201, China; 4 Animal Husbandry Research Institute, Kangding 626001, China; 5 Bureau of Agriculture, Animal Husbandry, Rural Affairs, and Science & Technology of Litang County, Litang County 627550, China; 6 College of Veterinary Medicine, Nanjing Agricultural University, Nanjing 210095, China; dywang@njau.edu.cn

**Keywords:** Ganzi Prefecture, yak, diarrhea, parasite, bacteria, virus

## Abstract

Yak diarrhea is a major health concern in high-altitude regions, yet data are lacking in Ganzi Tibetan Autonomous Prefecture, a key yak-producing area in China. This study aimed to describe the prevalence, mortality, seasonal patterns, and pathogen profiles of yak diarrhea in Ganzi Prefecture. Between 2017 and 2024, surveys were conducted across 5 counties, involving 5 cooperatives and 62 households. Fecal and blood samples were collected from healthy (*n* = 1805) and diarrheic yaks (*n* = 189) for pathogen detection. Among 1.9066 million yaks, breeding females accounted for 48.48% and calves 26.32%. Diarrhea prevalence was 12.22% in adult yaks and 20.91% in calves, with mortality rates of 15.71% and 28.29%, respectively. Cases peaked in spring and winter. Pathogen detection in healthy yaks revealed intestinal parasites (43.26%) and BVDV (2.33%). In diarrheic yaks, higher rates were observed for intestinal parasites (46.56%), *E. coli* (10.05%), *Salmonella* (10.58%), BVDV (22.75%), and *Theileria* (25.40%). Mixed infections were common. These findings provide a descriptive baseline for guiding region-specific control strategies.

## 1. Introduction

Yaks (*Bos grunniens*) are a unique bovine species adapted to high-altitude environments. They are primarily distributed across the Qinghai–Tibet Plateau and its surrounding mountainous regions, including China (Tibet, Qinghai, Sichuan, Gansu, Yunnan, and Xinjiang), as well as Mongolia, Nepal, Bhutan, India, Pakistan, and Afghanistan [1,2,3,4]. Globally, the yak population is estimated at approximately 15 million, with China accounting for over 90% [5]. In all these regions, yaks serve as a critical source of livelihood for pastoral communities, providing meat, milk, fiber, and draught power, and playing an essential role in high-altitude agricultural systems [6,7]. Yaks possess strong physiological traits such as cold and hunger tolerance, making them highly adapted to harsh environments [8]. Yak meat is rich in protein and low in fat, and is regarded as a high-quality, green animal protein source [9]. Ganzi Tibetan Autonomous Prefecture, located in Sichuan Province, China, at an altitude exceeding 3000 m, is one of the core yak-producing areas in the country [10]. The region possesses abundant yak resources and distinct regional characteristics [11]. However, due to the cold climate, complex terrain, and relatively low levels of farming management, the incidence of diarrhea among yaks in Ganzi is notably high [12].

Diarrhea is a major health challenge in cattle production worldwide, leading to significant economic losses due to reduced growth, treatment costs, and mortality [13]. In domestic cattle (*Bos taurus*), extensive research has established the etiological roles of viral pathogens, bacterial pathogens, and parasitic pathogens [14,15]. In recent years, epidemiological surveys have shown that various infectious and parasitic diseases are widespread among yak populations on the Qinghai–Tibet Plateau and in surrounding areas. Viral diseases such as bovine viral diarrhea, bovine tuberculosis, and nodular dermatitis have all been reported in yaks, and some outbreaks can lead to severe systemic lesions and high mortality rates [16,17,18,19,20]. In addition, parasitic infections—including liver flukes, *Theileria*, Babesia, and various intestinal protozoa—have been widely detected in yak populations in regions such as Qinghai, Gansu, and Tibet. Infection with these pathogens poses a persistent threat to the health and productivity of yaks [21,22,23,24].

However, in Ganzi Tibetan Autonomous Prefecture—the core yak-producing region of Sichuan Province—the overall prevalence of diseases in adult yaks and yak calves, as well as the pathogen spectrum and its distribution, have not yet been systematically described. To address this knowledge gap, we conducted field surveys in selected areas of Ganzi Tibetan Autonomous Prefecture from 2017 to 2024. The aim was to provide a descriptive baseline for the development of surveillance and prevention strategies tailored to the region, thereby promoting the sustainable development of the local yak industry.

## 2. Materials and Methods

### 2.1. Study Design

This was a descriptive field investigation conducted in Ganzi Tibetan Autonomous Prefecture, Sichuan Province, China, aiming to describe the farming structure, diarrhea prevalence, seasonal patterns, and pathogen profiles in yaks. Due to the logistical challenges inherent to high-altitude pastoral regions, including dispersed grazing systems, seasonal migration of herds, limited transportation accessibility, and harsh climatic conditions, a non-probability sampling approach was adopted. Purposive and convenience sampling strategies were used to maximize field accessibility and participation feasibility under real-world pastoral conditions. The study consisted of two components: (1) a cross-sectional survey of herd structure and retrospective recording of diarrhea incidence across selected counties, and (2) pathogen detection in fecal and blood samples collected from both healthy and diarrheic yaks. The investigation was conducted in two separate periods (2017–2019 and 2022–2024) based on project funding cycles and fieldwork feasibility. To ensure comparability between the two study periods, the same study regions, sampling framework, questionnaire structure, case definitions, and laboratory detection protocols were applied throughout both intervals whenever possible.

### 2.2. Study Population and Sampling

Target population: All yaks raised in Ganzi Tibetan Autonomous Prefecture. Study population: Yaks from five selected counties within Ganzi Prefecture, encompassing five cooperatives and 62 households. Sampling of counties: Counties were purposively selected based on geographical location and predominant farming system to represent diverse production settings. Specifically, two pastoral counties and three semi-agricultural and semi-pastoral counties were included. Sampling of cooperatives and households: Cooperatives and households were selected through convenience sampling. Inclusion criteria included willingness to participate, availability of basic herd records, and accessibility during the study period. A total of five cooperatives and 62 households were enrolled. Sampling of yaks: For the survey of herd structure and diarrhea incidence, all yaks within each selected cooperative or household were included (whole-herd sampling). For pathogen detection, yaks were selected based on clinical status at the time of sampling (healthy vs. diarrheic) according to the case definitions described below. No randomization was applied; samples were collected from all eligible animals encountered during farm visits. Because non-probability sampling was used, the study population may not fully represent all yak herds in Ganzi Prefecture. In particular, herds with better accessibility, stronger willingness to participate, or more complete records may have been overrepresented, potentially introducing selection bias. Therefore, the findings should be interpreted as descriptive epidemiological data rather than population-level estimates.

### 2.3. Case Definitions

Diarrhea in yaks: Defined as the presence of loose or watery feces for two or more consecutive days, with or without accompanying clinical signs such as depression, reduced appetite, or dehydration. This definition was applied uniformly for both incidence recording and sample collection. Diarrhea-associated mortality: Defined as death occurring during an episode of diarrhea, as reported by the herd owner or confirmed by study personnel. Mortality rate was calculated as the number of deaths attributed to diarrhea divided by the total number of diarrhea cases. Healthy yaks: Defined as animals with no clinical signs of diarrhea, normal fecal consistency, and normal feeding behavior at the time of sampling.

### 2.4. Data Collection on Herd Structure and Diarrhea

From 2017 to 2019 and again from 2022 to 2024, questionnaire surveys were conducted to collect data on yak population structure across all 18 counties (or county-level cities) in Ganzi Prefecture. The survey covered cooperatives, large-scale farms, and individual households. For detailed diarrhea monitoring, two pastoral counties and three semi-agricultural and semi-pastoral counties were selected from the 18 counties. Through structured questionnaire surveys and face-to-face household interviews conducted from 2019 to 2020, data were collected on the incidence and mortality of diarrhea in adult yaks and yak calves across these five counties. All questionnaire responses were verified when possible by cross-referencing with available farm records. Because some epidemiological information relied on retrospective reporting by herd owners, recall bias may have occurred.

### 2.5. Sample Collection

Between 2019 and 2020, 1805 fresh fecal samples were collected from clinically healthy yaks across 18 counties in Ganzi Prefecture. Among these, 1075 fecal samples were used for intestinal parasite examination, while 730 fecal samples were used for viral pathogen detection. In addition, 777 rectal swab samples were collected separately from healthy yaks for *Salmonella* detection. From 2020 to 2021, an additional 189 fecal samples were obtained from yaks exhibiting diarrhea according to the case definition above. All fecal samples were collected between 7:00 and 8:00 AM. Fecal samples from healthy yaks were collected immediately after spontaneous defecation from the ground using sterile polyethylene gloves, and only the inner portion of freshly deposited feces was sampled to minimize environmental contamination. For diarrheic yaks, loose or watery feces were collected directly from the rectum whenever possible, or immediately after defecation using sterile disposable tools. Approximately 50–100 g of fecal material was transferred into self-locking bags or EP tubes and appropriately labeled. Additionally, from 2019 to 2020, 777 anal swab samples were collected from healthy yaks using sterile saline-moistened swabs inserted approximately 6–7 cm beyond the anal sphincter. Swabs were rotated, withdrawn, and placed into PBS-containing EP tubes. From 2020 to 2022, 189 whole blood samples were collected from diarrheic yaks into anticoagulant vacuum tubes. Blood samples were collected from the jugular vein by trained veterinarians using sterile disposable needles and vacuum blood collection tubes. Prior to blood collection, the skin over the venipuncture site was cleaned and disinfected with 75% ethanol to minimize contamination and prevent local infection. Although some sampling areas were revisited during different field investigations, the sampled yaks were not the same individuals in each sampling session. All samples were transported on ice and stored at −20 °C until analysis.

### 2.6. Detection of Intestinal Parasites

Approximately 20 g of each fecal sample was taken and mixed with 1 to 2 volumes of deionized water using a glass rod to prepare a suspension. The mixture was filtered through a 60-mesh standard sieve into a 500 mL beaker to remove debris, then filled up with deionized water, thoroughly mixed, and left to stand for 1 h. The supernatant was discarded, and the sediment was retained for further analysis. For centrifugation, 2 to 3 volumes of deionized water was added to the sediment and transferred into a 10 mL centrifuge tube, followed by centrifugation at 2500 rpm for 3 min. The supernatant was carefully removed, and the pellet was resuspended with 2 to 3 drops of deionized water. One drop of the suspension was placed on a slide, covered with a coverslip, and examined under 10× and 40× objectives using light microscopy. The remaining suspension was mixed with 8 mL of saturated salt solution and centrifuged again at 2500 rpm for 3 min. The tube was then filled to the brim with saturated salt solution, creating an upward meniscus on which a coverslip was placed to contact the liquid surface. After standing for 10–15 min, the coverslip was carefully removed and examined under 10× and 40× objectives. Samples were considered positive if parasite bodies, eggs, oocysts, or larvae were observed by microscopy.

### 2.7. Detection of Escherichia Coli

Fecal samples suspended in buffered peptone water (BPW) were labeled and inoculated onto MacConkey agar or eosin methylene blue (EMB) agar plates using a sterile inoculation loop following standard bacterial isolation procedures. Plates were streaked using the three-line method and incubated at 37 °C for 18–24 h to observe colony morphology. Based on typical growth characteristics (metallic sheen on EMB and red colonies on MacConkey), approximately three suspected colonies were selected and inoculated into aliquoted LB enrichment broth, and then incubated on a shaker for about 12 h until reaching the logarithmic growth phase. The turbidity of the bacterial culture was visually assessed to confirm adequate bacterial growth. Under a Class II biosafety cabinet, approximately 1 mL of the enriched bacterial culture was transferred into sterile microcentrifuge tubes and centrifuged at 12,000 rpm for 5 min. The supernatant was discarded, and the bacterial pellet was resuspended in sterile nuclease-free water. The suspension was then heated at 100 °C for 10 min to lyse bacterial cells, followed by immediate cooling on ice and centrifugation at 12,000 rpm for 5 min. The resulting supernatant containing bacterial DNA was collected and used as the PCR template. PCR primers used were PHOA-F: GGCAATACACTCACTATGCGCTG and PHOA-R: AGGATTCGCAGCATGATCCTG. The primers were designed based on conserved sequences of the phoA gene retrieved from the NCBI GenBank database using Primer-BLAST (https://www.ncbi.nlm.nih.gov/tools/primer-blast/, accessed on 1 April 2026), and primer specificity was preliminarily evaluated by BLAST analysis. Primers were synthesized commercially by Sangon Biotech (Shanghai, China). The PCR reaction mixture (20 μL total volume) consisted of 10 μL of 2× PCR Mix, 1 μL each of forward and reverse primers, 2 μL of bacterial template, and nuclease-free water to volume. The PCR cycling conditions were: initial denaturation at 95 °C for 5 min, followed by 35 cycles of 95 °C for 15 s, 50 °C for 20 s, and 72 °C for 40 s, with a final extension at 72 °C for 10 min. Ten microliters of the PCR products were electrophoresed on a 10 g/L agarose gel, and the bands were visualized and documented.

### 2.8. Detection of Salmonella

Suspected *Salmonella* strains were isolated and cultured on selective media following the procedure outlined in GB4789.4-2016 [25] “Microbiological Examination of Food— *Salmonella* Testing.” Initial colonies were obtained and used for further analysis. Suspected colonies were inoculated into tetrathionate brilliant green broth (TTB) and incubated at 37 °C for 18 h. Total DNA was extracted from the enriched cultures using the phenol–chloroform method and used as a template for PCR detection. PCR amplification targeted a 284 bp fragment using the primers forward 5′-GTGAAATTATCGCCACGTTCGGGCAA-3′ and reverse 5′-TCATCGCACCGTCAAAGGAACC-3′, following the Sichuan provincial standard “PCR Detection Method for *Salmonella* from Yaks” (DB51/T 1828-2014) [26]. The 25 μL reaction mixture contained 12.5 μL of premix enzyme, 1 μL each of forward and reverse primers (10 μmol/μL), 2 μL of template DNA, and 8.5 μL of nuclease-free water. The thermal cycling conditions were: initial denaturation at 94 °C for 5 min, and 30 cycles of 94 °C for 45 s, 60 °C for 45 s, and 72 °C for 40 s, followed by a final extension at 72 °C for 10 min. PCR products were analyzed by electrophoresis on a 1.5% agarose gel, visualized, and documented. Raw sequence chromatograms were manually inspected for quality, and low-quality base calls at both ends were trimmed before sequence assembly and downstream BLAST analysis. Ambiguous nucleotide positions were checked based on bidirectional sequencing results to ensure sequence accuracy.

### 2.9. Virus Detection

Approximately 0.2 g of yak fecal sample was mixed with PBS buffer at a ratio of 1:5, followed by three freeze–thaw cycles at −80 °C. The mixture was centrifuged at 4000 rpm for 15 min using a refrigerated centrifuge, and the supernatant was then centrifuged at 12,000 rpm for 10 min. The resulting supernatant was filtered through a 0.22 µm filter, and 400 µL of the filtrate was used for RNA extraction. RNA was extracted using the Trizol method and immediately reverse transcribed into cDNA according to the instructions of the reverse transcription kit to prevent RNA degradation. The synthesized cDNA was aliquoted and stored at −20 °C for further analysis. RT-PCR was performed to detect bovine rotavirus (BRV), bovine coronavirus (BCoV), nebovirus (NEV), and bovine viral diarrhea virus (BVDV). The primer sequences and parameters were as follows: BRV, forward primer CCACCAGGTATGAATTGGAC, reverse primer CGCCATCTGAGTGATTACTC, product length 231 bp, annealing temperature 52 °C. BCoV: forward primer CGCCATCTGAGTGATTAC, reverse primer CGCCATCTGAGTGATTACTC, product length 230 bp, annealing temperature 49 °C. NEV: forward primer CGCTCCGTGTGGGATCACGA, reverse primer GCACGGGCTTCTTCTAGAGA, product length 194 bp, annealing temperature 56 °C. BVDV: forward primer GCCATGCCCTTAGTAGGACT, reverse primer CACCCTATCAGGCTGTRTYC, product length 230 bp, annealing temperature 61 °C. Each 25 µL PCR reaction mixture contained 12.5 µL of TaKaRa Ex Taq enzyme, 1.0 µL each of forward and reverse primers, 2 µL of cDNA template, and 8.5 µL of nuclease-free water. The PCR cycling conditions were as follows: initial denaturation at 94 °C for 4 min; 35 cycles of 94 °C for 30 s, annealing at the primer-specific temperature for 30 s, and extension at 72 °C for 30 s; a final extension at 72 °C for 8 min; and storage at 4 °C. PCR products were analyzed by electrophoresis on 1.5% agarose gels. Positive PCR products were sent to Shenggong Bioengineering for bidirectional sequencing. For all PCR and RT-PCR assays, positive controls and negative controls were included in each run to ensure assay validity and to monitor potential contamination. Quality control methods are the same as in Section 2.8.

### 2.10. Babesia Detection

Total DNA was extracted from 150 μL of whole blood using the phenol–chloroform method, with samples stored at 2–8 °C in anticoagulant tubes prior to extraction. Nested PCR targeting the 18S rRNA gene of Babesia was performed. The primers used were as follows: outer primers F: GATAACCGTGCTAATTGTAGG and R: ATCGTCTTCGATCCCCTAACT, amplifying an 843 bp fragment; inner primers F: AATTGTAGGGCTAATACATGTTCG and R: GAAAACATCCTTGGCAAATGCTTTCGC, amplifying a 760 bp fragment. The first-round PCR reaction mixture contained 12.5 μL Premix Taq, 1 μL each of outer primers (10 μmol/μL), 2 μL DNA template, and 8.5 μL ddH_2_O. The cycling conditions were: initial denaturation at 94 °C for 3 min; 30 cycles of 94 °C for 30 s, 60 °C for 40 s, and 72 °C for 1 min; a final extension at 72 °C for 7 min. The second-round PCR used 12.5 μL Premix Taq, 1 μL each of inner primers, 1 μL first-round product, and 9.5 μL ddH_2_O, with cycling conditions of 95 °C for 4 min; 35 cycles of 94 °C for 45 s, 55 °C for 20 s, and 72 °C for 30 s; and a final extension at 72 °C for 10 min. PCR products from the second round were analyzed by 1.5% agarose gel electrophoresis. Positive Babesia 18S rRNA PCR products were sent for sequencing. Sequence alignment was performed using BLAST on NCBI, and phylogenetic trees were constructed with MEGA 7 software using the Neighbor-Joining method with 1000 bootstrap replicates. Homology analyses were conducted with Lasergene 7.0 software to identify Babesia species based on evolutionary relationships and sequence similarity. Quality control methods are the same as in Section 2.8.

### 2.11. Ethical Approval

The animal study was reviewed and approved by the Animal Ethics Committee of Sichuan Minzu College (the study was approved under Ethics Approval Number ETH2025005 on 1 December 2016, and under Ethics Approval Number ETH2025006 on 1 January 2022). Written informed consent was obtained from the owners for the participation of their animals in this study.

### 2.12. Statistical Analysis

Comparisons of herd structure proportions between survey years were analyzed using the Chi-square test. Statistical analyses were performed using SPSS 27.0.1 (IBM Corp., Armonk, NY, USA). A value of *p* < 0.05 was considered statistically significant.

## 3. Results

### 3.1. Comparison of Yak Farming Structure Between 2019 and 2024

**Figure 1:**
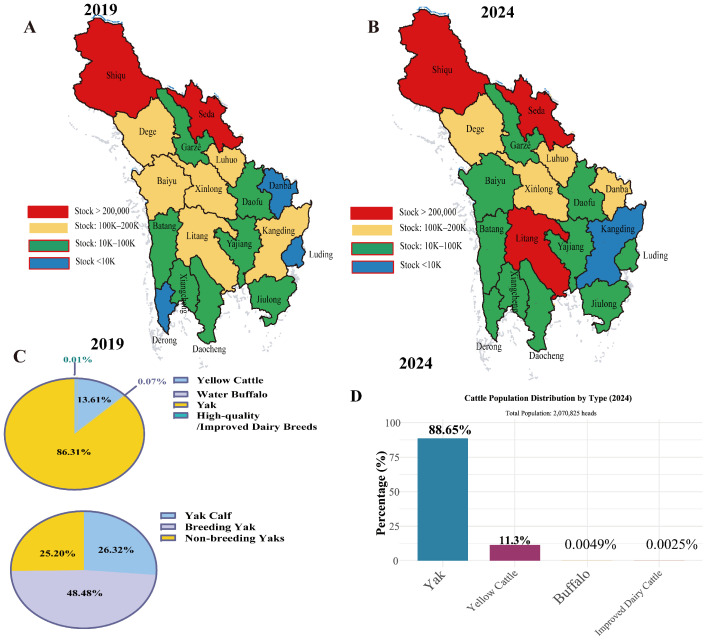
Spatiotemporal patterns of yak farming in Ganzi Tibetan Autonomous Prefecture (2019 vs. 2024). (**A**) Geographical distribution of yak population in 2019. (**B**) Geographical distribution of yak population in 2024. (**C**) Herd structure composition in 2019. (**D**) Herd structure composition in 2024.

A total of 1.9066 million yaks were recorded in Ganzi Prefecture during the survey period. Comparison of data from the final survey years (2019 and 2024) revealed changes in yak distribution across counties (Figure 1A,B). Herd sizes increased in some counties (e.g., Daofu and Yajiang) while decreasing slightly in others (e.g., Dege).

Reproductive sustainability improved overall, with multiple counties achieving breeding female ratios exceeding 50% in 2024—up from 40–45% in 2019. Litang and Batang counties showed breeding female proportions above 58%. Chi-square analysis indicated that the proportion of breeding females in the overall herd structure was significantly higher in 2024 than in 2019 (*p* < 0.05). Calf ratios showed considerable variability, reflecting divergent management outcomes across counties. The overall herd structure consisted of 48.48% breeding females, 26.32% calves, and 25.20% other yaks (Figure 1C,D). These results highlight progress in herd structure optimization despite regional disparities. Overall, these descriptive results suggest temporal changes in herd composition and management structure across the surveyed regions.

### 3.2. Temporal Changes in Yak Production Structure from 2017 to 2024

**Figure 2:**
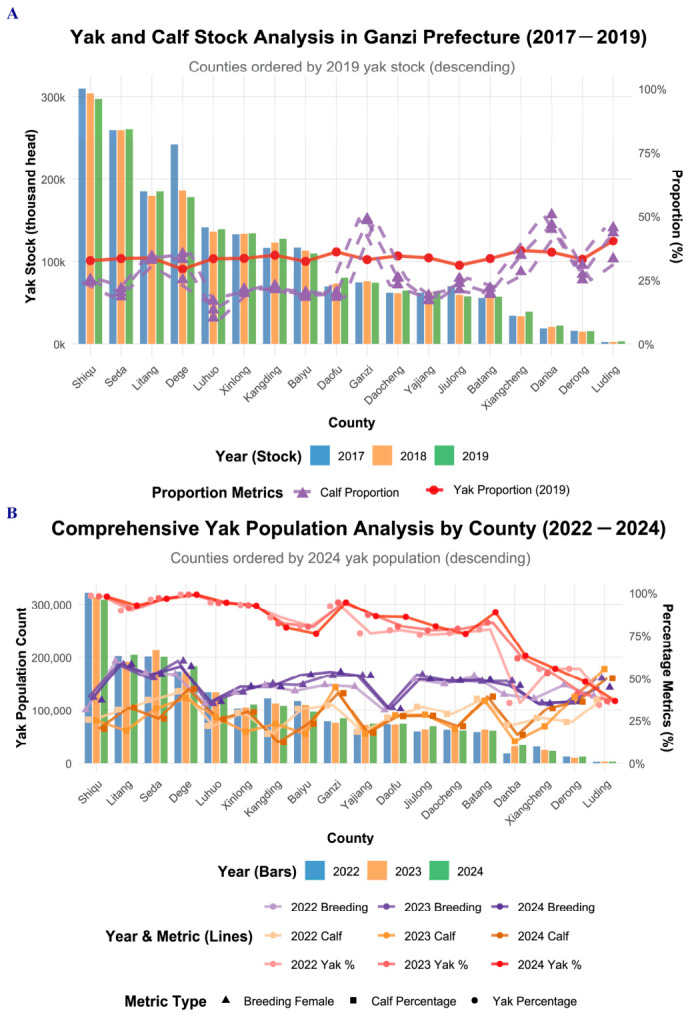
Investigation of Yak Farming in Ganzi Prefecture from 2017–2019 and 2022–2024. (**A**) Yak population dynamics in Ganzi Prefecture from 2017 to 2019. (**B**) Comprehensive yak population analysis from 2022 to 2024.

Distinct trends emerged between the two survey periods in terms of specialization and efficiency (Figure 2). From 2017 to 2019, the proportion of yaks within the total cattle inventory increased in most counties, signaling a shift toward specialization (Figure 2A). This trend continued and even intensified from 2022 to 2024, with several northern counties (e.g., Dege, Shiqu) maintaining yak proportions above 95% (Figure 2B).

The breeding female ratio showed consistent improvement across both periods, reflecting enhanced reproductive management. Calf ratios fluctuated more markedly during 2017–2019 but began to stabilize with higher baselines in the 2022–2024 cycle, suggesting better calf survival and herd renewal practices. Overall, these transitions indicate a strategic move from scale-oriented expansion to efficiency-focused production, with stronger specialization and improved herd dynamics over time.

### 3.3. Investigation of Diarrhea in Yak Calves in Ganzi Prefecture from 2019 to 2020

**Figure 3:**
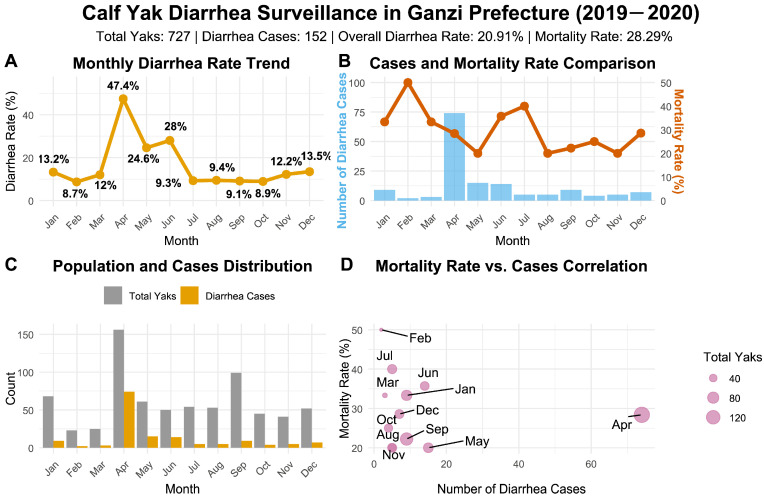
Survey results of diarrhea in Calf yak calves. (**A**) Monthly variation in diarrhea incidence. (**B**) Dual-axis chart showing both the number of diarrhea cases (bar graph) and the mortality rate (line graph). (**C**) Comparison between the total number of yak calves and diarrhea cases per month. (**D**) Scatter plot with bubble size representing the total number of yak calves.

Among 727 monitored calves, 152 exhibited diarrhea symptoms, yielding an overall diarrhea prevalence of 20.91%. Diarrhea occurrence showed distinct monthly variation, with the highest prevalence observed in April (47.44%) and the lowest in February (8.70%) (Figure 3A). Chi-square analysis demonstrated significant differences in diarrhea prevalence among different months (*p* < 0.05).

A total of 43 deaths were attributed to diarrhea, corresponding to a mortality rate of 28.29% among diarrheic calves. The highest mortality rate was recorded in February (50.00%), followed by July (40.00%). Notably, although April had the highest number of cases (*n* = 74), the mortality rate was relatively lower (28.38%), suggesting potentially different pathogenic factors or treatment response patterns in this month (Figure 3B). Mortality rates also showed significant monthly variation according to Chi-square analysis (*p* < 0.05). A positive correlation was observed between the total number of yak calves monitored and diarrhea cases across months (Figure 3C,D).

### 3.4. Investigation of Diarrhea in Adult Yaks in Ganzi Prefecture from 2019 to 2020

**Figure 4:**
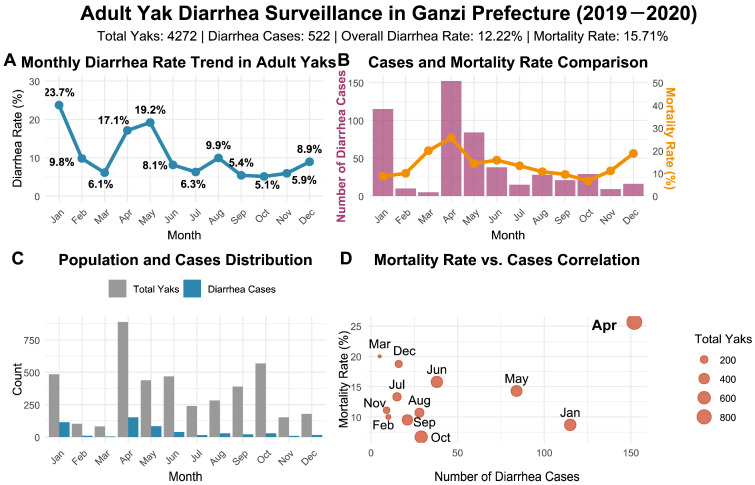
Epidemiological characteristics of diarrhea in adult yaks in Ganzi Prefecture. (**A**) Monthly variation in diarrhea incidence. (**B**) Dual-axis chart comparing the number of diarrhea cases (purple bars) and the mortality rate (orange line). (**C**) Distribution of observed yaks and diarrhea cases. (**D**) Scatter plot of mortality rate versus case number.

Among 4272 monitored adult yaks, 522 exhibited diarrhea symptoms, resulting in an overall diarrhea prevalence of 12.22% (Figure 4). Diarrhea occurrence displayed distinct seasonal characteristics, with prevalence peaking in January (23.71%) and remaining elevated in April (17.12%) and May (19.18%), while the lowest prevalence occurred from September to November (5.11–5.92%) (Figure 4A). Chi-square analysis demonstrated significant differences in diarrhea prevalence among different months (*p* < 0.05).

A total of 82 deaths were recorded, yielding a mortality rate of 15.71% among diarrheic adults. April had the highest number of deaths (*n* = 39) and a relatively high mortality rate (25.66%), while March showed a lower case number but a high mortality rate of 20.00% (Figure 4B). Mortality rates also differed significantly among months according to Chi-square analysis (*p* < 0.05). Compared with yak calves, adult yaks exhibited lower overall incidence and mortality rates, but demonstrated more pronounced seasonal patterns (Figure 4C,D).

### 3.5. Asymptomatic Carriage of Common Diarrheal Pathogens in Healthy Yaks

**Figure 5:**
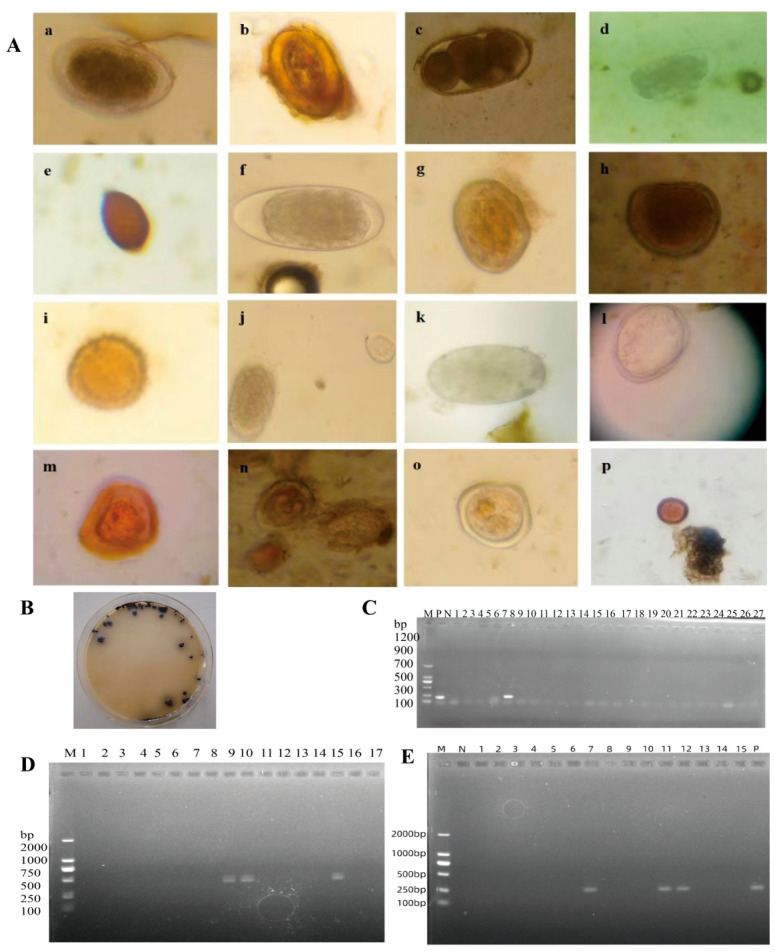
Detection of Common Pathogens in Fecal Samples from Healthy Yaks. (**A**) Microscopic morphology of partial parasite eggs and oocysts (10 × 40 magnification), showing: (**a**–**i**) nematode eggs; (**j**) a mixed field of nematode eggs and coccidian oocysts; (**k**,**l**) trematode eggs (fluke eggs); (**m**) cestode eggs (tapeworm eggs); and (**n**–**p**) coccidian oocysts. (**B**) Typical colony morphology of suspected *Salmonella* strains on BS agar medium, showing black colonies with metallic sheen. (**C**) Electrophoresis results of PCR detection for partial *Salmonella* isolates. Note: M: DNA Marker 1200 bp; P: Positive control; N: Negative control. (**D**) Electrophoresis results of RT-PCR detection for BRV (bovine rotavirus). (**E**) Electrophoresis results of RT-PCR detection for BVDV (bovine viral diarrhea virus).

Microscopic examination revealed various parasite egg morphologies, including nematode eggs, trematode eggs, cestode eggs, and coccidian oocysts, suggesting possible mixed infections of multiple parasite species (Figure 5A).

Characteristic black colonies with metallic sheen were observed on BS agar plates, and pink colonies with or without black centers were observed on XLD selective media (Figure 5B). confirmed *Salmonella* identification was confirmed by representative PCR electrophoresis bands (Figure 5C). Representative RT-PCR electrophoresis results for BRV and BVDV are shown in Figure 5D and 5E, respectively.

**Figure 6:**
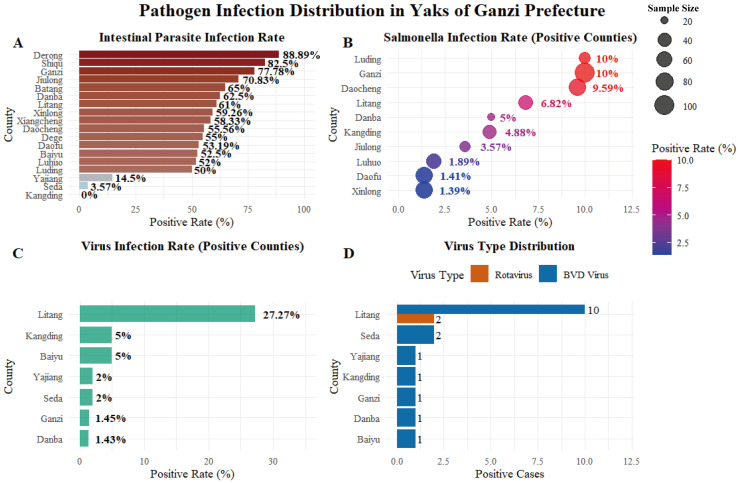
Asymptomatic Carriage of Common Diarrheal Pathogens in Yak. (**A**) Intestinal parasite infection. (**B**) *Salmonella* infection rate. (**C**) Virus infection rate. (**D**) Virus type distribution.

Overall, from a data perspective, among 1075 fecal samples examined for intestinal parasites from healthy yaks in Ganzi Prefecture, the overall positive rate for intestinal parasite eggs was 43.26% (465/1075), indicating widespread subclinical parasite carriage within the healthy yak population (Figure 6A), among the 777 rectal swab samples collected for *Salmonella* detection, 30 samples tested positive, yielding a positivity rate of 3.86% (Figure 6B). For viral infection detection, 19 out of 730 fecal samples examined for viral pathogens tested positive, yielding an overall positivity rate of 2.60% (Figure 6C). Litang County had the highest rate (27.27%). Among them, the detection rate for bovine rotavirus (BRV) was 0.27% (2/730), and for bovine viral diarrhea virus (BVDV) it was 2.33% (17/730) (Figure 6D).

In summary, a high prevalence of intestinal parasite carriage was observed in healthy yaks in Ganzi Prefecture. Although the detection rates of *Salmonella* and viruses were relatively low, the presence of these pathogens indicates potential subclinical carriage in apparently healthy animals. This highlights the need for enhanced monitoring and disease control measures to safeguard herd health.

### 3.6. Detection of Pathogens in Diarrheic Yaks

**Figure 7:**
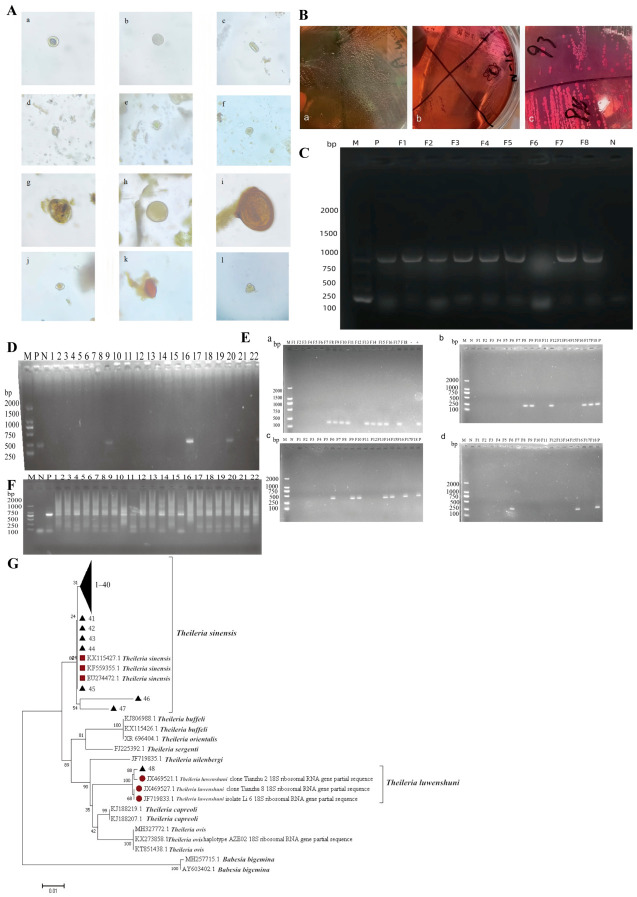
Diarrhea yak pathogen infection situation. (**A**) Map of partial egg morphology (10 × 40); (**a**–**c**,**g**,**h**,**k**): nematode eggs; (**d**–**f**,**j**): coccidian oocysts; (**i**): trematode eggs; (**l**): cestode eggs. (**B**) Colony morphology of *E.coli;* (**a**,**b**): EMB agar (black with metallic sheen); (**c**): MacConkey agar (pink). (**C**) Electrophoresis diagram of PCR detection of *E.coli.* (**D**) Electrophoresis diagram of PCR detection of *Salmonella.* (**E**) Electrophoresis diagram of RT-PCR detection of BVDV (**a**), BCoV (**b**), BRV (**c**), and NEV (**d**). (**F**) Electrophoresis diagram of PCR detection of *Piroplasma.* (**G**) Phylogenetic tree based on the 18S rRNA sequence of *Piroplasma.* Note: M: DL 2000 DNA Marker; N: Negative control (deionized water); P: Positive control; F1–F15: Yak blood samples from diarrhea yaks.

A total of 189 fecal and blood samples from diarrheic yaks were analyzed. Overall, 177 samples tested positive for at least one detected pathogen, yielding an overall positivity rate of 93.65%. Intestinal parasite eggs were detected in 46.56% (88/189) of samples, with representative egg morphologies (nematode eggs, coccidian oocysts, trematode eggs, and cestode eggs) shown in Figure 7A. *Escherichia coli. (E. coli)* colonies appeared with a black metallic sheen on eosin methylene blue agar and as pink, smooth, neatly edged colonies on MacConkey agar (Figure 7B). Nineteen samples tested positive (10.05%), with representative PCR electrophoresis results shown in Figure 7C. *Salmonella* was detected in 20 samples, with a positivity rate of 10.58% (Figure 7D). Viral detection rates were as follows: BVDV 22.75% (43/189), BCoV 4.23% (8/189), BRV 3.17% (6/189), and NEV 1.05% (2/189). Representative RT-PCR electrophoresis results for BVDV, BCoV, BRV, and NEV are shown in Figure 7E. For Piroplasma (*Theileria*), in blood samples, *Theileria* spp. were detected in 25.40% (48/189), with *Theileria orientalis* accounting for 97.91% (47/48) and *Theileria luwenshuni* for 2.08% (1/48). Representative PCR electrophoresis results are shown in Figure 7F, and the phylogenetic tree based on 18S rRNA sequences is presented in Figure 7G.

**Figure 8:**
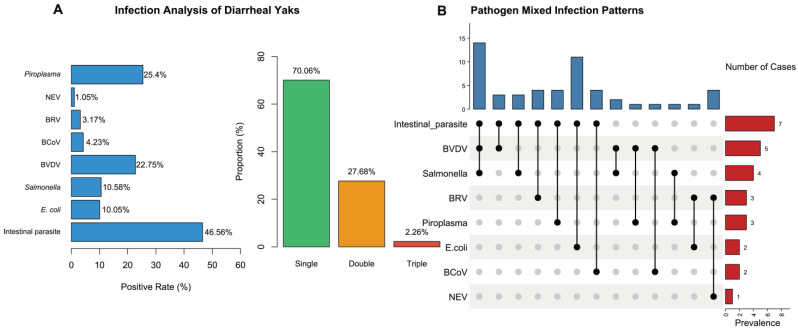
Infection analysis of diarrheal yaks. (**A**) Pathogen Infection rate. (**B**) Pathogen mixed infection patterns.

Mixed infections: Among the 177 positive samples, single-pathogen infections accounted for 70.06% (124/177), while mixed infections accounted for 29.94% (53/177) (Figure 8A). Chi-square analysis showed that single-pathogen infections were significantly more frequent than mixed infections (*p* < 0.05). Among mixed infections, dual infections represented 27.68% and triple infections 2.26% (Figure 8A). Dual infections occurred significantly more frequently than triple infections according to Chi-square analysis (*p* < 0.05). The most frequently observed mixed infection patterns were intestinal parasites + *E. coli* (26.41% of mixed infections) and *Salmonella* + BVDV (20.75%) (Figure 8B). In addition to the major pathogens systematically tested, field observations indicated that diarrhea was also associated with Cryptosporidium infection, stress, and poor management practices.

In summary, multiple pathogens were widely detected in diarrheic yaks, with complex infection patterns primarily involving intestinal parasites, *E. coli*, *Salmonella*, BVDV, and *Theileria* spp. These findings highlight the need for strengthened pathogen monitoring and integrated control strategies.

## 4. Discussion

This descriptive field investigation provides an overview of yak diarrhea in Ganzi Tibetan Autonomous Prefecture. The findings indicate that diarrhea is prevalent year-round with seasonal peaks in spring and winter, particularly affecting calves (prevalence 20.91%) more than adult yaks (12.22%). Pathogen detection revealed a wide range of infectious agents in both healthy and diarrheic yaks, with intestinal parasites being the most commonly detected. Mixed infections, especially intestinal parasites combined with *E. coli* or *Salmonella* with BVDV, were frequently observed in diarrheic animals.

Ganzi Prefecture is an important yak breeding region in China, with a well-structured herd and traditional free-grazing systems [10,11]. The high detection rate of intestinal parasites in healthy yaks (43.26%) may reflect the widespread use of traditional grazing systems, where yaks frequently come into contact with contaminated water sources and environmental pathogens, while deworming programs are inconsistently implemented. The seasonal pattern of diarrhea, with peaks in spring and winter, likely relates to the region’s high-altitude ecological conditions. Cold winter temperatures may favor the survival and transmission of bacterial and viral pathogens, while spring coincides with increased parasitic egg-laying activity [12,22]. This seasonal pattern is consistent with observations in Himalayan yaks, where infectious diseases exhibit clear seasonal cyclicity [27]. Additionally, forage quality and availability are lowest during winter and early spring, potentially leading to reduced energy intake and weakened immunity, which may increase susceptibility to diarrhea [8].

Compared with healthy yaks, diarrheic yaks showed higher detection rates for intestinal parasites (46.56% vs. 43.26%), *Salmonella* (10.58% vs. 3.86%), BVDV (22.75% vs. 2.33%), and BRV (3.17% vs. 0.27%), while *E. coli*, BCoV, and NEV were detected exclusively in diarrheic yaks in this sample. These findings indicate that the presence of these pathogens was more frequently associated with diarrheic yaks than with healthy yaks. However, causal relationships between pathogen detection and diarrhea cannot be established based on this descriptive study. The BVDV detection rate in diarrheic yaks (22.75%) aligns with the high seroprevalence (84.52%) reported in Qinghai yaks [16], indicating widespread BVDV circulation in the Qinghai–Tibet Plateau region. A previous meta-analysis also confirmed that BVDV is prevalent in yaks across mainland China [28]. A recent review further confirmed that BVDV infection rates are increasing in yak populations across multiple provinces, including Sichuan [17]. The detection of LSDV in yaks from both India and China [19,20] further underscores that yaks are susceptible to multiple viral pathogens that can cause systemic disease and digestive lesions.

In blood samples, *Theileria* spp. were detected in 25.40% of diarrheic yaks in this study. This prevalence is generally consistent with previous reports indicating that piroplasma infections are widely distributed among yak populations across different regions of China [22,23,29,30], although regional variation in prevalence has been reported [31]. These differences may reflect geographic and ecological variation in tick-borne pathogen distribution across the Qinghai–Tibet Plateau. Similarly, intestinal parasites were frequently detected in diarrheic yaks, supporting previous studies showing that parasitic infections are common in yak populations in western China [21,24]. Together, these findings suggest a substantial burden of parasitic and tick-borne pathogens in yaks from Ganzi Prefecture. In Ganzi Prefecture itself, a recent study reported Cryptosporidium spp. and Giardia intestinalis in yaks with molecular detection rates of 7.2% and 15.7%, respectively [11]. A meta-analysis estimated the overall prevalence of Cryptosporidium in yaks across China at 6.9% [32], with regional variation: in Qinghai yaks, Cryptosporidium prevalence ranged from 1.8% to 12.5% [33]. Previous studies have also reported that fungal and protozoan parasites are commonly associated with diarrhea in yaks [29,34]. Bacterial enteropathogens are also important causes of diarrhea in yaks. In addition to *E. coli* and *Salmonella*, Shigella sonnei has been isolated from diarrheic yaks, with a prevalence of 3.9% in a previous study [35]. The observation of mixed infections, including intestinal parasites combined with *E. coli* and *Salmonella* with BVDV, is consistent with the complex infection patterns reported in other yak populations [14,22,23]. In dairy calves, mixed infections with Cryptosporidium, Giardia, and bacteria are also commonly associated with diarrhea [36,37], further supporting the importance of mixed infections in diarrheal diseases.

Notably, although April had the highest number of cases (*n* = 74), the mortality rate was relatively lower (28.38%), whereas February showed the lowest prevalence but the highest mortality rate. This pattern may reflect the harsher environmental conditions during winter, including low temperatures, nutritional stress, and delayed access to treatment in remote pastoral areas, which could increase disease severity and mortality among affected calves.

Mixed infections were observed in approximately 30% of diarrheic yaks, with dual infections being the most common pattern. The frequent co-occurrence of intestinal parasites with *E. coli*, and *Salmonella* with BVDV, suggests that pathogen–pathogen interactions may contribute to disease severity and complicate clinical management. However, whether these mixed infections directly cause more severe outcomes, or simply reflect high background exposure levels, cannot be determined from the current data.

Several limitations should be considered when interpreting the findings of this study. First, the use of purposive and convenience sampling may have introduced selection bias, as herds with better accessibility and stronger willingness to participate were more likely to be included. As a result, the prevalence estimates reported in this study may not fully reflect the epidemiological situation of the entire yak population in Ganzi Prefecture. Second, the two study periods (2017–2019 and 2022–2024) were discontinuous and were conducted under different field conditions and project schedules. Therefore, observed temporal differences should be interpreted cautiously and should not be considered direct longitudinal trends. Third, the case definitions for diarrhea and mortality were based on farmer reports and clinical observations, which may be subject to recall bias and inter-observer variability. This may have influenced the accuracy of reported diarrhea incidence and mortality data. Fourth, due to the descriptive and cross-sectional nature of the study, no causal inferences can be drawn regarding the role of specific pathogens in disease development. Fifth, the sample size for diarrheic yaks (*n* = 189) was relatively small compared with the healthy yak sample, which may limit the precision of prevalence estimates for less common pathogens. Sixth, non-infectious causes of diarrhea (e.g., nutritional factors, stress, management practices) were not systematically assessed, although they were noted anecdotally during field observations. The absence of these data may limit comprehensive interpretation of the multifactorial nature of diarrhea in yaks. It should also be noted that parasites were categorized only by morphology into major groups and not identified to the genus level, which is a limitation of this study. Future studies employing longitudinal cohort designs, probability sampling, standardized diagnostic criteria, and inclusion of non-infectious risk factors are warranted to validate and extend these findings.

Despite these limitations, the findings offer descriptive baseline data that may inform region-specific surveillance and prevention strategies. The high prevalence of intestinal parasites in both healthy and diarrheic yaks suggests that parasite control programs (e.g., regular deworming) should be prioritized. The detection of multiple viral pathogens, including BVDV and BCoV, indicates that vaccination strategies could be considered. Furthermore, the frequent observation of mixed infections highlights the need for integrated control approaches rather than pathogen-specific interventions. The high detection rates of tick-borne pathogens (*Theileria*) in this study, consistent with reports from other yak-producing regions [22,23,30,31], also emphasize the importance of tick control measures in the region.

## 5. Conclusions

This study systematically examined the prevalence, seasonal patterns, and etiological basis of yak diarrhea in Ganzi Prefecture. Diarrhea was common in both adult yaks and calves, with higher prevalence and mortality observed during spring and winter. Multiple pathogens, including intestinal parasites, Piroplasma, *E. coli*, *Salmonella*, BVDV, BCoV, BRV, and NEV, were detected in diarrheic yaks, and mixed infections were frequently observed. These findings provide baseline epidemiological data for improving disease surveillance and developing region-specific prevention and control strategies for yak diarrhea in Ganzi Prefecture.

## Data Availability

The data presented in this study are available on request from the corresponding author.
